# Updates on the Association Between Anemia and Heart Failure: A Systematic Review

**DOI:** 10.7759/cureus.69101

**Published:** 2024-09-10

**Authors:** Hyder Mirghani, Atheer A Alshreef, Hammad A Al-Temani, Najla K Alanazi, Aseel Algohani, Wejdan M Alrshidi, Norah A Alturki, Abdulaziz Turki Alqabli, Fares M Alruwaili, Ghadeer S Almarwni

**Affiliations:** 1 Internal Medicine, University of Tabuk, Tabuk, SAU; 2 Medical School, University of Tabuk, Tabuk, SAU

**Keywords:** anemia, association, cardiovascular disease, heart failure, outcomes, prevalence, prognosis

## Abstract

This systematic review aims to investigate the potential relationship between anemia and heart failure (HF) by summarizing existing literature on the topic. A comprehensive search was performed using four major databases, PubMed, Scopus, Web of Science, and ScienceDirect, to find the relevant literature. Ten studies, including a total of 2,828 participants, with 1,451 (51.3%) males, were included in this review. Iron deficiency anemia was the most prevalent type in the included studies; however, two studies included megaloblastic anemia. The prevalence of anemia in patients diagnosed with HF ranged from 33.3% to 69.8%, with a total prevalence of 1,643 (58.1%). Hypertension, diabetes mellitus, chronic kidney disease, and atrial fibrillation were the most commonly associated comorbidities in patients with HF. Anemia patients had a considerably higher risk of mortality than those without anemia. Anemia served as a marker of disease severity rather than an independent predictor of death in congestive individuals. Anemia was substantially correlated with elevated serum creatinine, left ventricular hypertrophy, and left atrial enlargement. According to the findings of this review, anemia has a significant impact on the prognosis of HF. In patients with HF, anemia may be a reliable indicator of both short- and long-term all-cause mortality as well as the rates of all-cause HF events. Future and ongoing research may provide vital information that may help guide clinical judgments in the future.

## Introduction and background

Anemia and heart failure (HF) are prevalent medical diseases that frequently coexist and are recognized to significantly affect a patient’s overall health and quality of life. Anemia is defined as a reduction in the quantity of hemoglobin or red blood cells in the blood, whereas HF is the result of the heart’s inability to pump enough blood to meet the body’s requirements. Numerous studies have examined the relationship between anemia and HF, and the results suggest that the two disorders are frequently related and may even make one another worse [1].

Anemia lowers the blood’s ability to carry oxygen, which lowers the quantity of oxygen that reaches the heart and other tissues and organs. This is one of the main causes of the link between anemia and HF. The heart may have to work harder as a result to pump enough oxygen-rich blood to the body, which could strain it more. This increased strain over time may have a role in the onset or aggravation of HF [2].

Moreover, research indicates that up to half of all HF patients also have anemia, demonstrating the prevalence of anemia as a comorbidity in HF patients. Given that anemia has been linked to several unfavorable outcomes in HF patients, such as a higher risk of hospitalization, lower quality of life, and an increased mortality rate, the high incidence of anemia in HF patients is a cause for concern [3].

Apart from the effects anemia may have on HF patients, HF itself may also play a role in the emergence of anemia. Reduced renal function from chronic HF may lead to a reduction in erythropoietin production, which is a hormone that promotes the synthesis of red blood cells. Anemia may eventually arise from this decrease in the quantity of red blood cells in the blood [4].

Healthcare professionals should actively screen for and treat anemia in patients with HF because of the potential effects that anemia may have on these patients. Depending on the underlying etiology of the anemia and the specifics of each patient, treatment options for anemia in HF patients may include iron supplementation, erythropoiesis-stimulating medications, or blood transfusions [5,6].

Anemia and HF have a complicated and varied relationship, with the potential for both disorders to worsen one another and have detrimental effects on individuals. To maximize patient outcomes and enhance quality of life, healthcare professionals should be watchful when it comes to identifying and treating anemia in patients with HF. Although there are anecdotal reports indicating a possible connection between HF and anemia, thorough and methodical studies investigating this relationship are scarce. This study seeks to address this knowledge gap. This study aims to systematically review the existing literature on the association between anemia and HF to provide a comprehensive analysis of the current evidence and identify areas for further research.

The study aims to assess the prevalence of anemia in patients diagnosed with HF and to examine how anemia affects the progression and management of HF. Additionally, the review seeks to uncover potential mechanisms that may explain the relationship between anemia and HF. Finally, the study provides recommendations for clinical practice and suggests directions for future research in this field.

## Review

Methodology

In this systematic review investigating the connection between anemia and HF, we followed the Preferred Reporting Items for Systematic Reviews and Meta-Analyses (PRISMA) guidelines [7]. An extensive electronic search was performed across databases such as PubMed, Web of Science, Scopus, and ScienceDirect to identify pertinent research published in English. Specific terms such as “anemia,” “iron deficiency,” “heart failure,” and “cardiovascular risk” were used in the search strategy. Each reviewer independently sorted through the search results, selected pertinent papers, collected information, and graded the quality of the included research using the proper assessment tools.

To qualify for inclusion in this review, studies needed to be randomized controlled trials, cohort studies, case-control studies, or cross-sectional studies investigating the relationship between anemia and HF in human subjects. These studies needed to be published in English and include clear diagnostic criteria for both anemia and HF. Additionally, they needed to report relevant outcomes, such as the prevalence, prognosis, or impact of anemia on heart failure. Only studies conducted within the last year (2023-2024) were considered for inclusion.

The review excluded case reports, editorials, commentaries, or reviews that did not present original data. Animal studies, in vitro research, and studies involving non-human subjects were also disregarded, as any studies not directly related to the association between anemia and HF. Furthermore, studies not published in English or those lacking sufficient data regarding the relationship between anemia and HF were not included.

Data Extraction

Rayyan (QCRI) was used to confirm the accuracy of the results [8]. Using the inclusion and exclusion criteria, the titles and abstracts found during the literature search were assessed for relevancy. The study team carefully reviewed all papers that satisfied the inclusion criteria. Any disagreements were resolved through consensus. A predetermined data extraction form was used to record important study details, such as titles, authors, location, publication year, demographics, gender, prevalence rates of anemia in HF patients, follow-up duration, type of anemia, mean hemoglobin level, associated comorbidities, and outcomes. To evaluate the risk of bias, a third-party assessment method was used.

Data Synthesis

For a qualitative evaluation, summaries of the research findings and their components were developed using information gathered from relevant studies. Once the data collection phase for the systematic review was completed, the most effective approach for utilizing the data from these studies was determined.

Risk of Bias Assessment

The study quality was assessed using the critical assessment criteria for studies reporting prevalence data developed by the Joanna Briggs Institute (JBI) [9]. There were nine questions in this instrument, and a score of 1 was given for a positive response and a score of 0 for a negative, ambiguous, or irrelevant response. Low, moderate, and high quality were assigned to scores that fell between 4 and 7, and 8 and above, accordingly. Disagreements were settled through conversation after researchers evaluated the study quality independently.

Results

**Figure 1:**
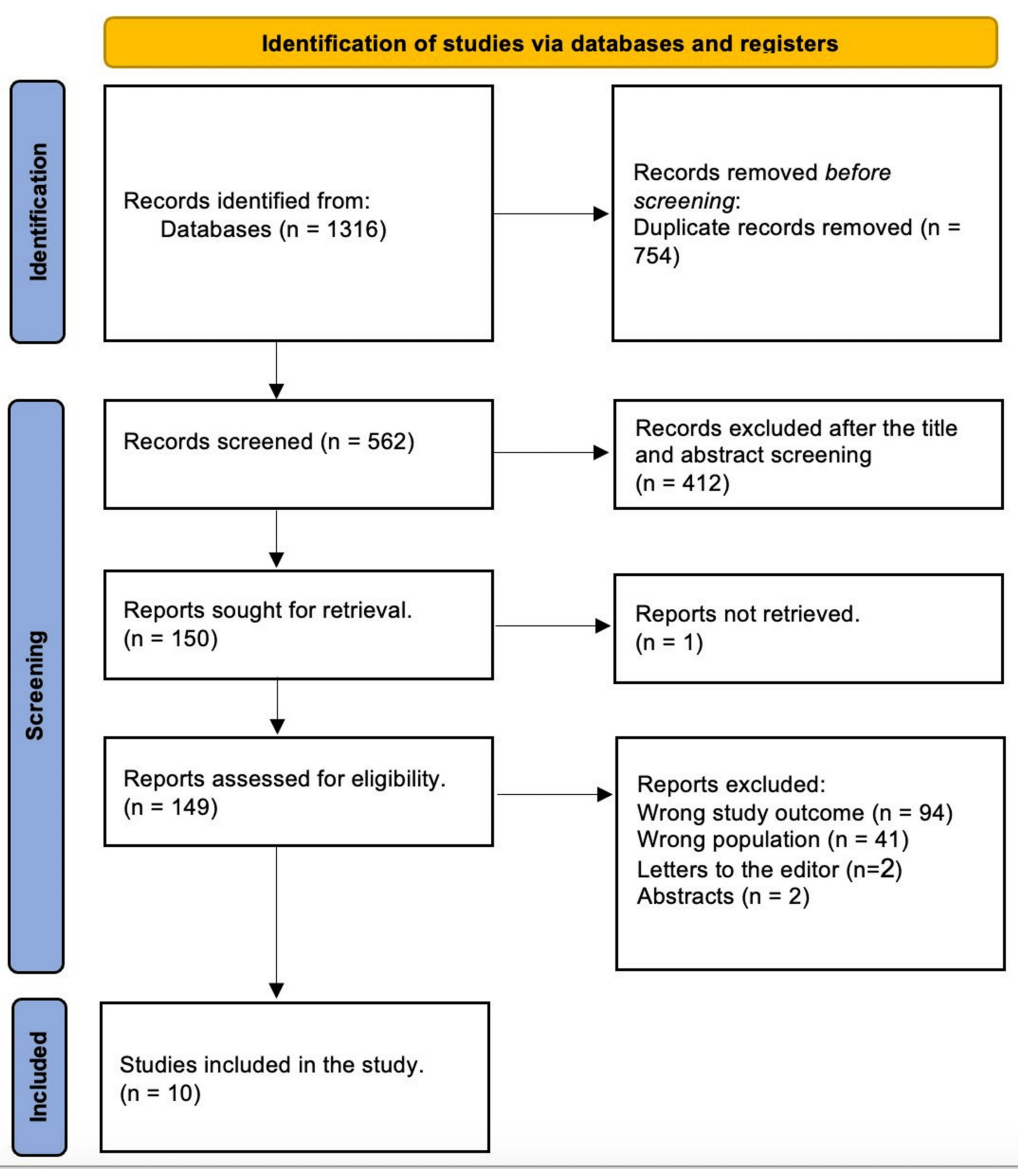
Preferred Reporting Items for Systematic Reviews and Meta-Analyses (PRISMA) flow diagram illustrating the study selection.

After 754 duplicates were removed, a total of 1,316 studies were found through a systematic search. After 562 studies had their titles and abstracts evaluated, 412 papers were discarded. Only one article was not located out of the 150 reports that were required to be retrieved. Subsequently, 149 articles passed the screening process for full-text evaluation; 94 were rejected due to incorrect study results, 41 due to incorrect population type, two articles were letters to the editor, and two were abstracts. Finally, 10 studies satisfied the requirements for eligibility. An overview of the procedure used for study selection is illustrated in Figure 1.

Sociodemographic Features of the Included Studies

The sociodemographic information of the included studies is displayed in Table 1. Ten studies, including a total of 2,828 participants [10-19], with 1,451 (51.3%) males, were included in our review. Five studies were retrospective cohorts [10-12,17,19], four were cross-sectional [14-16,18], and only one was a prospective observational study [13]. Four studies were conducted in India [13,14,17,19], one in Italy [10], one in Turkey [11], one in Japan [12], one in Cameroon [15], one in Peru [16], and one in Congo [18].

**Table 1:** The sociodemographic attributes of the included studies. NM: not mentioned

Study ID	Study design	Country	Participants	Mean age	Males (%)
Scicchitano et al., 2023 [10]	Retrospective cohort	Italy	434	75 ± 11	226 (52%)
Köseoğlu & Özlek, 2024 [11]	Retrospective cohort	Turkey	212	70.6 ± 10.5	96 (45.3%)
Naito et al., 2023 [12]	Retrospective cohort	Japan	238	76 ± 8	101 (42.4%)
Qureshi et al., 2024 [13]	Prospective observational	India	388	58.8	229 (59%)
Bista et al., 2023 [14]	Cross-sectional	India	100	65.4 ± 12.5	52 (52%)
Djibrilla et al., 2024 [15]	Cross-sectional	Cameroon	86	59.4 ± 17.7	31 (46.4%)
Tirado et al., 2023 [16]	Cross-sectional	Peru	1003	NM	513 (51.4%)
Das et al., 2023 [17]	Retrospective cohort	India	96	59.9 ± 12.7	67 (69.8%)
Ikama et al., 2023 [18]	Cross-sectional	Congo	171	57.5 ± 16.5	79 (46.2%)
Shrivastava et al., 2023 [19]	Retrospective cohort	India	100	64.5	57 (57%)

Clinical Outcomes

Most of the included studies did not compare or separate acute HF and chronic HF while studying the prevalence and effects of anemia. Iron deficiency anemia (IDA) was the most prevalent type in the included studies; however, two studies included megaloblastic anemia [14,17]. The prevalence of anemia in patients diagnosed with HF ranged from 33.3% [18] to 69.8% [17], with a total prevalence of 1,643 (58.1%).

Hypertension, diabetes mellitus (DM), chronic kidney disease (CKD), and atrial fibrillation (AF) were the most commonly associated comorbidities in patients with HF. Patients with anemia had a considerably higher risk of mortality than those without anemia. There was a correlation between anemia and B-type natriuretic peptide (BNP), renal function, increased left cardiac remodeling, and increased plasma volume expansion. Anemia served as a marker of disease severity rather than an independent predictor of death in congestive individuals. Anemia was substantially correlated with elevated serum creatinine, left ventricular hypertrophy, and left atrial enlargement.

Scicchitano et al. (2023) [10] reported a prevalence of anemia of 59% in patients with acute HF and chronic HF, specifically noting that anemia is often associated with IDA. Similarly, Köseoğlu and Özlek (2024) [11] found that 38.2% of patients suffering from chronic HF had anemia, indicating a significant burden carried by this population. Furthermore, Das et al. (2023) [17] reported that 69.8% of participants had anemia, of which a striking 65.7% were specifically iron deficient.

Bista et al. (2023) [14] noted that nearly 65% of HF patients had IDA, emphasizing a consistent pattern across studies. Scicchitano et al. noted that patients with anemia had a significantly higher risk of mortality compared to those without anemia, even when accounting for complications such as renal function issues and BNP levels, a peptide whose concentration is often elevated in HF. Köseoğlu and Özlek further elucidated this point by showing that patients with IDA had a relative risk of death 3.5 times higher than those without, and anemic individuals had a risk nearly five times higher than controls. This suggests that while anemia serves as a marker of disease severity, it may also indicate a wider spectrum of pathophysiological processes affecting these patients. In addition, Naito et al. (2023) [12] found that anemic patients experienced not only worse renal function but also elevated cardiac remodeling, indicated by increased plasma volume expansion and poor nutritional status.

Djibrilla et al. (2024) [15] reported that while anemia did correlate with elevated serum creatinine levels and left ventricular hypertrophy, it did not consistently link to unfavorable outcomes. Ikama et al. (2023) [18] noted that anemia commonly coexists with other significant health issues such as hypertension and renal insufficiency.

Table 2 summarizes the clinical features of the included studies. Table 3 summarizes the results of a critical appraisal using the JBI tool for several studies, indicating their inclusion in the systematic review. Each study’s answers to appraisal questions (Q1 to Q9) are displayed, with “Y” for yes, “U” for unclear, “N” for no, and “NA” for not applicable. Notably, all studies are marked as included, suggesting they met the criteria despite some having unclear responses or non-applicable elements.

**Table 2:** Clinical features and results of the included studies. HF: heart failure; Hb: hemoglobin; AHF: acute heart failure; CHF: heart failure; DM: diabetes mellitus; CKD: chronic kidney disease; CAD: coronary artery disease; COPD: chronic obstructive pulmonary disease; AF: atrial fibrillation; BNP: B-type natriuretic peptide; ID: iron deficiency; IDA: iron deficiency; HFpEF: heart failure with preserved ejection fraction; HFrEF: heart failure with reduced ejection fraction; NYHA: New York Heart Association; NM: not mentioned

Study ID	Type of HF	Type of anemia	Follow-up (days)	Mean Hb (mg/dL)	Prevalence of anemia	Associated comorbidities	Main outcomes
Scicchitano et al., 2023 [10]	AHF/CHF	IDA	NM	13 ± 2	256 (59%)	AF (43%), CKD (33%), CAD (31%), DM (24%), COPD (21%)	Patients with anemia had a considerably higher risk of mortality than controls. There was a correlation between anemia and BNP and renal function. Anemia served as a marker of the severity of the disease rather than an independent predictor of death in congestive individuals
Köseoğlu & Özlek, 2024 [11]	CHF	IDA	66.2 ± 12.1	11.9 ± 1.8	81 (38.2%)	Hypertension (76.9%), ID (50.9%), AF (39.2%), DM (31.1%), CKD (11.8%)	ID affects at least 50% of individuals with HFpEF, while anemia affects more than 33%. Patients with ID had a relative risk of death roughly 3.5 times higher than that of anemic individuals, who had a relative risk that was nearly five times higher
Naito et al., 2023 [12]	AHF/CHF	IDA	NM	10.9 ± 1.2	112 (47.1%)	Hypertension (81%), AF (32%), DM (23%), CAD (22%), COPD (10%)	Compared to patients without anemia, anemic HFpEF patients had worse nutritional status, worse renal function, increased left cardiac remodeling, and increased plasma volume expansion
Qureshi et al., 2024 [13]	CHF	IDA	6	NM	274 (71%)	NM	Individuals with HFrEF had a high burden of anemia and iron insufficiency, with ID being more prevalent. An independent predictor of composite outcomes, such as cardiac death or rehospitalization, is iron deficiency
Bista et al., 2023 [14]	AHF/CHF	IDA and megaloblastic anemia	NM	NM	65 (65%)	DM (41%), hypertension (34%), COPD (16%), CAD (45%), AF (3%)	IDA was the most prevalent kind of anemia, affecting nearly two-thirds of HF patients. Anemia was more common in females and COPD patients
Djibrilla et al., 2024 [15]	AHF	NM	NM	12.3 ± 2.4	37 (42.7%)	Hypertension (57.4%), DM (13.3%), CKD (7.4%), AF (2.9%)	HF patients in hospitals frequently suffer from anemia. Anemia was substantially correlated with elevated serum creatinine, left ventricular hypertrophy, and left atrial enlargement. However, anemia was not linked to unfavorable results
Tirado et al., 2023 [16]	AHF/CHF	NM	NM	NM	630 (62.8%)	NM	In the Peruvian population, anemia raises the risk of dying from congestive HF
Das et al., 2023 [17]	AHF/CHF	IDA and megaloblastic anemia	NM	10.1 ± 2.5	67 (69.8%)	Hypertension (73.9%), DM (66.7%)	Anemia was observed in 69.8% of patients, with 65.7% having ID. In the anemic group, 71.6% had ID, whereas (34.5%) the non-anemic group was also iron deficient. A higher prevalence of ID was found in females, patients with more severe disease, and those having lower ejection fraction
Ikama et al., 2023 [18]	AHF/CHF	IDA	48.7 ± 15.4	9.4 ± 1.5	57 (33.3%)	NM	A common comorbidity among people with HF is anemia. Low hemoglobin rate, NYHA class III-IV, longer hospitalization stay, rehospitalization rate, mortality rate, renal insufficiency, and hypertension are frequent among anemic patients diagnosed with HF and predict disease severity
Shrivastava et al., 2023 [19]	AHF	IDA	NM	NM	64 (64%)	NM	Anemia and cardiovascular disorders were statistically significantly associated. Patients who were smokers had high blood pressure or had DM had a higher risk of anemia

**Table 3:** JBI critical appraisal tool. JBI: Joanna Briggs Institute; Y: yes; U: unclear; N: no; NA: not applicable

Study ID	Q1	Q2	Q3	Q4	Q5	Q6	Q7	Q8	Q9	Decision
Scicchitano et al., 2023 [10]	Y	Y	Y	Y	Y	Y	Y	Y	Y	Included
Köseoğlu & Özlek, 2024 [11]	Y	Y	Y	Y	NA	Y	Y	Y	Y	Included
Naito et al., 2023 [12]	Y	Y	Y	Y	Y	Y	Y	Y	Y	Included
Qureshi et al., 2024 [13]	Y	Y	Y	Y	NA	Y	Y	Y	Y	Included
Bista et al., 2023 [14]	Y	Y	Y	Y	NA	Y	Y	Y	Y	Included
Djibrilla et al., 2024 [15]	Y	Y	Y	Y	NA	Y	Y	Y	Y	Included
Tirado et al., 2023 [16]	Y	Y	Y	Y	NA	Y	Y	Y	Y	Included
Das et al., 2023 [17]	U	Y	Y	Y	NA	Y	Y	Y	Y	Included
Ikama et al., 2023 [18]	Y	Y	Y	Y	Y	Y	Y	NA	Y	Included
Shrivastava et al., 2023 [19]	Y	Y	Y	Y	NA	U	Y	Y	Y	Included

Discussion

Patients with HF are more likely to have mixed anemia, of which the two most significant pathophysiological processes are iron shortage and inadequate erythropoietin production, according to a substantial body of clinical and fundamental research data [20]. In this review, most included studies did not compare or separate acute HF and chronic HF while studying the prevalence and effects of anemia. Moreover, we did not include the subgroup analysis for types of HF and anemia which may be a source of bias.

We found that the prevalence of anemia in patients diagnosed with HF ranged from 33.3% [18] to 69.8% [17], with a total prevalence of 58.1%. These rates are similar to those reported by Kyriakou et al. (14-71%) [21]. The lack of a common definition might readily account for the wide range of anemia prevalence values. The length of follow-up in each trial and the variations in patient characteristics provide an additional explanation.

The following factors are linked to the higher frequency of anemia in HF patients [20,22-25]: reduced iron intake caused by (1) inadequate meal intake and hypothalamic appetite regulatory dysfunction and (2) gastrointestinal mucosa irritation following antiplatelet or anticoagulant medication treatment, which results in microscopic blood flow loss in the gastrointestinal tract. Simultaneously, edema of the intestinal mucosa during the advanced phase of HF prevents iron absorption. (3) Patients with HF have higher plasma levels of inflammatory agents such as interleukin-6 (IL-6) and tumor necrosis factor-alpha (TNF-α). The gastrointestinal tract’s ability to absorb iron is ultimately decreased by IL-6, which increases the production of hepcidin protein, which, in turn, inhibits membrane transporter protein-1 in hepatocytes, macrophages, and the gastrointestinal tract. (4) Inflammatory components TNF-α and IL-6 activate the transcription factors GATA-binding protein 2 and the nuclear factor κ light chain enhancer of activated B cells, which, in turn, prevent renal erythropoietin production. (5) Hemodilution. Additionally, the growth and differentiation of erythroid progenitor cells in the bone marrow are directly inhibited by these factors.

IDA was the most prevalent type in the included studies in this review; however, two studies included megaloblastic anemia [14,17]. Prior studies have shown that absolute iron deficiency typically persists, patients with acute HF often have a combined iron shortage, and absolute iron deficiency raises the risk of early rehospitalization in acute HF patients [26,27]. Ferric carboxymaltose has the potential to lower the rehospitalization risk; however, as the initial studies did not report the patients’ iron deficiency status or the effects of anemia and iron deficiency treatments, it is not feasible to infer the impact of iron deficiency status on the prognosis of acute HF [28].

We found that hypertension, DM, CKD, and AF were the most commonly associated comorbidities in patients with HF. These two circumstances coexisting can be explained by a pathophysiological process. Reduced endogenous erythropoietin synthesis from renal failure may eventually result in anemia. The cardio-renal syndrome is caused by an increase in cardiac hemodynamic load as a result. We might also explain our results for both systolic and diastolic blood pressure using this approach [21].

This study found that patients with anemia had a considerably higher risk of mortality than those without anemia. A 3% increase in the risk of death is linked to a 1% drop in hematocrit, according to a study by Mozaffarian et al. [29]. Similarly, Teng et al. [30] provided evidence that a 7% decrease in mortality occurrences corresponds to every 1 g/dL increase in hemoglobin levels. Regarding re-admission, Felker et al. [31] found that there is an estimated 12% increase in the risk of death or re-hospitalization within 60 days for every 1 g/dL decrease in hemoglobin. Unfortunately, it is not possible to determine whether the stage of anemia (hemoglobin level) increases the risk of death or morbidity from the data we were able to extract. Thus, we can conclude that the more severe the anemia, the worse the prognosis, by measuring the risk of mortality based on hemoglobin levels. Our findings align with published research. More precisely, anemia is linked to major consequences for hospitalized in-patients with HF according to Young et al. [32]. Individuals with hemoglobin levels below 10 g/dL have an increased risk of death or readmission, as well as lengthier hospital stays.

## Conclusions

The current review highlights the significant association between anemia and prognosis in acute HF. Anemia emerges not only as a common comorbidity in patients with HF but also as a potential prognostic marker. The evidence suggests that anemia tends to correlate with an increased risk of both short- and long-term all-cause mortality in these patients. Furthermore, it appears to be associated with elevated rates of all-cause HF events, which underscores the need for clinicians to assess hemoglobin levels as part of routine patient evaluations.

Recognizing the link between anemia and negative outcomes in acute HF could facilitate more personalized treatment approaches that target both issues. Future research is essential to better understand how anemia affects the progression of HF and its complications. Studies should aim to identify effective interventions for managing anemia in this group, as addressing anemia could enhance patient prognosis. Exploring the effectiveness of various treatments, such as iron supplementation and erythropoiesis-stimulating agents, will be important for improving patient outcomes.
